# LGBTQI cancer patients’ quality of life and distress: A comparison by gender, sexuality, age, cancer type and geographical remoteness

**DOI:** 10.3389/fonc.2022.873642

**Published:** 2022-09-20

**Authors:** Jane M. Ussher, Kimberley Allison, Janette Perz, Rosalie Power, The Out with Cancer Study Team

**Affiliations:** Translational Health Research Institute, School of Medicine, Western Sydney University, Sydney, NSW, Australia

**Keywords:** cancer, LGBTQI, distress, quality of life, minority stress, intersectionality, discrimination, transgender

## Abstract

**Background:**

There is growing acknowledgement of the psycho-social vulnerability of lesbian, gay, bisexual, transgender, queer and/or intersex (LGBTQI) people with cancer. The majority of research to date has focused on cisgender adults with breast or prostate cancer.

**Study Aim:**

This study examined psycho-social factors associated with distress and quality of life for LGBTQI cancer patients and survivors, across a range of sexualities and gender identities, intersex status, tumor types, ages and urban/rural/remote location using an intersectional theoretical framework.

**Method:**

430 LGBTQI people with cancer completed an online survey, measuring distress, quality of life (QOL), and a range of psycho-social variables. Participants included 216 (50.2%) cisgender women, 145 (33.7%) cisgender men, and 63 (14.7%) transgender and gender diverse (TGD) people. Thirty-one (7.2%) participants reported intersex variation and 90 (20%) were adolescents or young adults (AYA), aged 15-39. The majority lived in urban areas (54.4%) and identified as lesbian, gay or bisexual (73.7%), with 10.9% identifying as bisexual, and 10.5% as queer, including reproductive (32.4%) and non-reproductive (67.6%) cancers.

**Results:**

Forty-one percent of participants reported high or very high distress levels, 3-6 times higher than previous non-LGBTQI cancer studies. Higher rates of distress and lower QOL were identified in TGD compared to cisgender people, AYAs compared to older people, those who identify as bisexual or queer, compared to those who identify as lesbian, gay or homosexual, and those who live in rural or regional areas, compared to urban areas. Elevated distress and lower QOL was associated with greater minority stress (discrimination in life and in cancer care, discomfort being LGBTQI, lower outness) and lower social support, in these subgroups. There were no differences between reproductive and non-reproductive cancers. For the whole sample, distress and poor QOL were associated with physical and sexual concerns, the impact of cancer on gender and LGBTQI identities, minority stress, and lack of social support.

**Conclusion:**

LGBTQI people with cancer are at high risk of distress and impaired QOL. Research and oncology healthcare practice needs to recognize the diversity of LGBTQI communities, and the ways in which minority stress and lack of social support may affect wellbeing.

## 1 Introduction

There is growing acknowledgement of the psycho-social vulnerability and health disparities experienced by sexual and gender minority (SGM) people with cancer, who are lesbian, gay, bisexual, transgender, queer and/or intersex (LGBTQI) (1, 2). Epidemiological studies report that cisgender lesbian, gay and bisexual (LGB) women and men are at higher risk of anal, breast, gynecological and lung cancer in comparison to their heterosexual counterparts (3). There is also evidence emerging of higher cancer burden in transgender and gender diverse (TGD) people (4, 5), including those who reject a binary gender, or who report a gender identity that is different from sex assigned at birth. These disparities are partly explained by higher rates of smoking and alcohol consumption and low rates of cancer screening in LGBT communities (6, 7). Obesity and nulliparity are additional risk factors for lesbian and bisexual women, with anal sex and higher rates of HPV infection, as well as the impact of HIV, acting as risks factors for gay men (3) and TGD people (5). Exogenous hormone use as part of gender affirmation has also identified as a potential risk factor for cancer (8). In 2017, the American Society of Clinical Oncology recognized the needs of this “medically underserved” (3) population, concluding there is “insufficient knowledge about the health care needs, outcomes, lived experiences and effective interventions to improve outcomes” for LGBTQI populations.

### 1.1 Psycho-social vulnerability of LGBTQI people with cancer

Evidence of greater psycho-social vulnerability of SGM people with cancer is primarily based on research with white cisgender adults, predominantly with breast or prostate cancer (1). It has been reported that gay or bisexual cisgender men with prostate cancer report higher psychological and cancer-related distress and lower quality of life (9–14), in comparison to heterosexual men. Cisgender breast cancer survivors who identify as lesbian, bisexual or queer (LBQ), report higher levels of distress and lower quality of life than heterosexual women (1, 15, 16). There is also some evidence that LBQ women with gynecological cancer report significantly higher rates of depression, anxiety and post-traumatic stress disorder (17), than their heterosexual counterparts. A national survey including a range of cancer types reported higher rates of poor self-reported health in lesbian women and higher rates of psychological distress in bisexual women, compared to heterosexual women (16).

There are significant gaps in research on the psycho-social health of LGBTQI people with cancer. There is limited research on LGBTQI cancer across non-reproductive tumor streams, and on reproductive cancers other than breast and prostate cancer (1, 3). There is also little research on LGBTQI adolescent and young adult (AYA) experiences of cancer (18), other than two recent studies reporting higher rates of anxiety (19, 20) and depression (20) in LGBTQ AYAs, compared to non-LGBTQ adolescent and young adult (AYAs). There is little research on psycho-social outcomes of TGD people with cancer (1, 3, 21), other than a recent study reporting higher rates of depression in TGD compared to cisgender people (22), and small scale qualitative research studies of TGD cancer survivorship experiences (8, 23, 24). This is also little research including LGBTQ people of color, migrants, and Aboriginal and Torres Strait Islander people (3, 25). There is no research to date on the cancer experiences of people with intersex variations (1). Recent systematic literature reviews have concluded that research is needed to understand psycho-social outcomes and the complexity of LGBTQI experiences of cancer comparing across ages and SGM subgroups, including people who are TGD and intersex (1, 3, 6, 18). There is also a need to explore potential differences between reproductive and non-reproductive tumor types, given the absence of research on non-reproductive cancers (1, 3) and healthcare professional assumptions that LGBTQI status may not be relevant for these diagnoses (26). This is the aim of the present study. It has been recommended that any new research needs to recognize the diversity of LGBTQI communities and investigate how this diversity may affect cancer survivorship and wellbeing (6, 27). It has been suggested that an intersectional theoretical framework is the most appropriate way to meet these aims (28), through facilitating understanding of how the complex spheres of identity intersect and the ways that “multiple axes of oppression” (29) may affect health outcomes among LGBTQI people with cancer (1, 30).

### 1.2 Factors associated psycho-social vulnerability in LGBTQI people with cancer

Understanding the factors associated with psycho-social vulnerabilities identified within the LGBTQI cancer population is also essential, to ameliorate distress and inform the development of LGBTQI inclusive cancer care (1, 26). Concerns about sexual wellbeing, embodied change and intimate relationships are recognized to be a major source of distress in the general cancer population (31, 32). There is some evidence that gay men with prostate cancer report greater distress about changes to sexual (33), urinary, and bowel functioning (14, 34, 35), and greater sexual and ejaculatory bother (10, 36, 37), compared to heterosexual men. This is accompanied by anxiety about the impact of cancer on gay identity and relationships (11, 38–40), and lower masculine self-esteem (9, 35). Gay and bisexual men with prostate cancer have been reported to be less likely to be in an ongoing relationship than heterosexual men (9, 41), and to receive less affection from partners (35). However, there is some evidence that gay and bisexual men experience higher sexual functioning (14, 42), sexual confidence, and a greater likelihood to attempt sexual rehabilitation, in comparison with heterosexual men (42).

Distress in adult LBQ breast cancer survivors has been associated with greater social and relationship difficulties (43), and disruption in sexual activity and desire (44), in comparison to heterosexual women. Conversely, other research has reported lower levels of concern with sex and appearance and less disruption in sexual activity in lesbian and bisexual women with breast cancer (45–47), compared with heterosexual women. In one study, lesbian and bisexual women with breast cancer who had a woman partner had better physical and mental health than heterosexual women who were unpartnered, or with a male partner (48). There is a need for further research to examine changes to sexuality, physical embodiment, gender identity and LGBTQI identity in a broader range of intersecting LGBTQI identities and age groups.

Fear of cancer recurrence (FCR) is associated with anxiety, depression, and decreased quality of life in the general cancer population (49). There is some evidence of greater FCR in gay and bisexual men with prostate cancer, in comparison to heterosexual men (34, 50). Conversely, lower FCR was reported by lesbian women with breast cancer, in comparison with heterosexual women (51). Younger age has consistently been associated with greater FCR (49, 52), however, there is no research to date that has examined FCR in AYA LGBTQI people with cancer.

Minority stress, the chronic and cumulative stress on those with stigmatized sexual and gender identities (53, 54), has been put forward as an explanation for the high rates of distress reported in the general LGBTQI population (55–59), and as a factor contributing to distress in LGBTQI cancer survivors (1, 60). Minority stress includes stigma, social exclusion, and discrimination commonly associated with LGBTQI identities (described as distal stressors), as well as negative self-beliefs and expectations of LGBTQI people, including internalized homophobia, concealment of identity, and stigma consciousness – vigilance and expectation of rejection in social interactions (described as proximal stressors) (61, 62). There is evidence of an association between discrimination and anxiety, depression and poor physical health in LBQ breast cancer survivors (15, 53, 63). LBQ women with breast cancer who were more ‘out’ in disclosing their sexual identity in general life reported higher distress in one study (53). This may be the result of stigmatization and negative cancer health care professional reactions to patient disclosure of LGBTQI identity (2, 26, 64). In this vein, LBQ women with breast cancer (65), and gay men with prostate cancer (9) report lower satisfaction with cancer care than their heterosexual counterparts. Economic hardship, which can be a consequence of minority stress, has also been associated with distress in LBQ breast cancer survivors (53, 63). For LGBTQI individuals, minority stress potentially compounds the impact of other stressors associated with cancer diagnosis and treatment, including uncertainty of treatment outcome, fear of cancer recurrence, co-morbidity, and disease stage (53, 66, 67). The impact of minority stress, and other factors associated with distress and poor quality of life, across intersecting LGBTQI identities remains unexplored (3).

Social support can ameliorate the impact of sexual and relationship difficulties (37, 44), embodied change (68) and minority stress in the context of cancer (43, 69), resulting in better quality of life and functioning (70). Higher social support is also related to better psychological outcomes in LGBQ cancer populations (19, 71, 72). For older LGBTQI people, social support is often provided by ‘chosen family’ (73), which includes intimate partners and friends (43, 51), and through social connectedness with LGBTQI people (62, 74). Parental and sibling support and acceptance is of particular importance for younger LGBTQI people in relation to psychological wellbeing (75). However, some LGBTQI people experience low social support, due to not having an intimate partner (37), family rejection (76), or because of living in rural or remote areas where they feel isolated from other LGBTQI people (62) and impacted by stigma and social exclusion (77). The absence of social safety, reflected in low social support, has been described as the “missing piece” in understanding the impact of minority stress on the health of LGBTQI people (78). The association between social support and distress for LGBTQI cancer survivors requires further exploration, across intersecting identities, cancer types and geographical remoteness (3).

### 1.3 Research aims and questions

This exploratory cross-sectional study aims to address these gaps in the research literature by examining distress and quality of life for LGBTQI people with cancer, and a range of psycho-social factors reported to be associated with distress and quality of life, comparing sexuality and gender identities, intersex status, age groups, reproductive and non-reproductive tumor types and geographical remoteness (urban/rural/regional), using an intersectional theoretical framework.

Our research questions were:

1. For LGBTQI people with cancer, does distress and quality of life differ by gender, sexuality, intersex status, age, cancer type, or remoteness?
2. Do sexual concerns, physical concerns, impact of cancer on gender and LGBTQI identity, FCR, minority stress, and social support differ across gender, sexuality, age, intersex status, cancer type, or geographical remoteness?
3. Are sexual concerns, physical concerns, impact of cancer on gender and LGBTQI identity, FCR, minority stress, and social support associated with distress and quality of life for LGBTQI people with cancer?
4. Does this association differ across gender, sexuality, intersex status, age, cancer type, or geographical remoteness?

### 1.4 Summary of key acronyms

AYA, Adolescents and young adults

HCP, Health care professional

LBQ, Lesbian, bisexual and queer

LGB, Lesbian, gay and bisexual

LGBQ, Lesbian, gay, bisexual and queer

LGBT, Lesbian, gay, bisexual and transgender

LGBTQI, Lesbian, gay, bisexual, transgender, queer and/or intersex

SGM, Sexual and gender minority

TGD, Transgender and gender diverse

QOL, Quality of Life

## 2 Methods

### 2.1 Study design and theoretical framework

This study was part of a broader mixed methods project, the *Out with Cancer Study*, which explored LGBTQI experiences of cancer and cancer care from the perspectives of LGBTQI people with cancer, caregivers, and healthcare professionals (26, 60, 79). This paper presents the findings of an online survey completed by 430 LGBTQI people with cancer, examining the psycho-social factors associated with distress and quality of life (QOL).

The project adopts an intersectional theoretical framework, which acknowledges that all people inhabit multiple interconnected social identity categories, such as gender, sexuality, cultural background and age (80), and that these categories are embedded in systems of social stratification, associated with inequality or power (81–83). An intersectional perspective recognizes that identity cannot be reduced to the summary of social groups to which a person belongs; rather, attention is paid to how social identities intersect to produce a meaningful whole in a way that cannot be explained by looking at one social identity alone (82). These categories are properties of individuals in terms of their identities, as well as characteristics of social contexts, and influence social practices and health and wellbeing (84). Whilst intersectionality theory has predominantly been used in qualitative research designs (81), it can also inform quantitative research by informing research questions and analysis that acknowledges the multiplicative effects of identity positions (85). We are adopting a *both/and* framework (29, 82), which considers *both* the “master category” of LGBTQI identity *and* the “subordinate categories” (29, 82) of age, TGD status, sexuality, intersex status, ethnicity and cultural background, geographical remoteness, and type of cancer. While these subordinate categories and identities are analyzed separately in statistical analyses, the “emergent effects” that occur when multiple identities intersect is interpreted through an intersectional lens (82).

The project was guided by principles of integrated knowledge translation (iKT) (86), with a stakeholder advisory group (comprising LGBTQI people with cancer and carers, cancer HCPs, and representatives from LGBTQI health and cancer support organizations) involved at all stages. The study received ethics approval from Western Sydney University Human Research Ethics Committee (ref. no. H12664), with secondary approval from the ACON (formerly the AIDS Council of New South Wales) (ref. no. 2019/09).

### 2.2 Participants and recruitment

Participants were eligible for this study if they: (a) identified as LGBTQI; (b) had been diagnosed with cancer or had undergone a medical intervention related to cancer risk; and (c) were at least 15 years old. The study was advertised on social media (Facebook, Twitter, Instagram), *via* cancer and LGBTQI community organizations (including partner organizations), through cancer research participation databases, and at in-person LGBTQI events and cancer support groups. Participants were also encouraged to share the survey link with others who might be eligible for participation. Participant demographics were monitored and recruitment strategies were refined through the data collection period with the aim of increasing the recruitment of underrepresented groups. The survey was open from September 2019 to September 2021.

### 2.3 Measures

The survey comprised a series of closed and open-ended measures, with questions tailored for: (a) people who were lesbian, bisexual or queer (LGBQ); (b) people who were TGD; and (c) people who had an intersex variation. Participants could choose which version of the survey to complete and could complete more than one pathway. Closed-ended questions presented in this paper are described below. Open-ended questions are presented in additional publications (26, 60).

#### 2.3.1 Distress

Psychological distress was measured using the ten-item Kessler Psychological Distress Scale (K10) (87), which asked participants to rate how frequently they have experienced various distressing feelings over the past 30 days. Participants responded using a five-point Likert scale (*none of the time* – *all of the time*) and scores on individual items were summed to produce a total distress score ranging from 10 to 50. Scores were categorized as indicating low (10-15), moderate (16-21), high (22-29) or very high (30-50) distress in accordance with Australian Bureau of Statistics guidelines (88). In this study, the K10 had excellent internal consistency (Cronbach’s α=.926).

#### 2.3.2 Quality of life

A single item derived from the EORTC-QLQ-C30 (89), which is widely used as a QOL scale in cancer research (90), asked participants to rate their overall QOL over the past week using a seven-point Likert scale (1 = *very poor* – 7 = *excellent*).

#### 2.3.3 Sexual concerns

Eleven items from the EORTC Sexual Health Questionnaire [EORTC SHQ-C22 (91, 92)] were used to assess sexual health. The EORTC was adapted to remove gendered designations of questions (“for men/women only”) to be inclusive of TGD and intersex bodies; to remove items overlapping with other sections of our survey; and to assess sexual issues both before *and* after cancer. Participants were asked to rate the extent to which they experienced sexual satisfaction and concern before and after cancer, using a four-point Likert scale (*not at all* – *very much; N/A*s excluded). Sexual concerns were operationalized as a decrease in satisfaction scores or an increase in concern scores from pre- to post-cancer. The total number of sexual concerns reported was then calculated (range 0-11).

#### 2.3.4 Physical concerns

Fourteen items assessed the presence and extent of concerns with changes to the body related to cancer. These were adapted from a previous survey on prostate cancer in gay/bisexual men (9), with modifications made to be inclusive of the broader LGBTQI cancer population. Participants reported the extent to which they were concerned with potential bodily changes using a four-point Likert scale (*not at all* to *very much*). Responses were dichotomized as no concern (*not at all*) or some concern (*a little/quite a bit/very much*). The total number of physical concerns reported was then calculated (range 0-14).

#### 2.3.5 Impacts of cancer on LGBTQI identity

Three items were developed based on the format of the Illness Intrusiveness Ratings Scale (IIRS) (93) to assess the impact of cancer on feelings about being LGBTQI, openness about being LGBTQI, and involvement with LGBTQI communities. These questions were asked separately about for sexuality, TGD identity, and intersex variations, with responses averaged for participants who completed this item for more than one identity. Participants responded using a four-point Likert scale (1 = *not at all* to*–* 4 = *very much*; *N/A*s excluded), with scores summed to produce a total impact score (range 3-12, higher scores indicating greater impact). Cronbach’s alpha for the three items was .571.

#### 2.3.6 Impact of cancer on gender identity

A single item was developed to assess the impact of cancer on feelings on gender identity, based on the format of the IIRS (93) and the content of items on masculinity/femininity from the EORTC-SHQ-C22 (91, 92). Participants responded using a four-point Likert scale (1 = *not at all* to 4 = *very much*) to assess whether cancer has impacted on their ‘feelings about gender identity (e.g. as a man, woman, transgender, non-binary or gender fluid person)’.

#### 2.3.7 Fear of cancer recurrence

A single item from the unidimensional FCR4 and FCR7 scales (94) was used to assess the extent to which participants were afraid their cancer may recur over the past week. Participants responded using a five-point Likert scale (1 = *not at all –* 5 = *all the time*).

#### 2.3.8 Minority stress

Ten items measuring distal and proximal aspects of minority stress were identified through review of existing LGBTQI minority stress measures (95–97), described below.

##### 2.3.8.1 Discrimination in general life and cancer care

A single item based on a previous study of sexual minority breast cancer survivors (43) was adapted to ask “have you experienced discrimination for being LGBTQI in your life in general?” (asked in separate survey pathways for LGBQ, TGD and intersex participants as relevant). A second item was added to assess experiences of discrimination “as part of your cancer care”. Response options were modified to use a four-point Likert scale (1 = *not at all* to 4 = *very much*), consistent with other measures in the survey.

##### 2.3.8.2 Discomfort in being LGBTQI

Three items assessing comfort, concealment and feelings about LGBTQI identity were selected from existing LGBT minority stress and identity measures (95–97). Participants were asked to report their agreement to statements about being “comfortable being LGBTQI”, “keep[ing] careful control over who knows you are LGBTQI” (concealment motivation), and if they “wish they were not LGBTQI” (internalized prejudice). All questions were asked using separate wording for LGBQ, TGD and intersex participants (e.g., focusing on sexuality, TGD or intersex status). Responses were made using a five-point Likert scale (*strongly disagree* to– *strongly agree*); after reverse coding for some items, scores were summed to produce a total minority stress score (range 3-15, with higher scores indicating greater minority stress). Cronbach’s alpha for the three items was .655.

##### 2.3.8.3 Outness to others

The 5-item disclosure subscale of the Nebraska Outness Scale (98), measuring details of disclosure and concealment of LGBTQI identities, was adapted for use in this study. The “strangers” item was replaced with “healthcare professionals”, and the response scale was changed from percentages to *none/a few/some/most/all* to be consistent with other survey items. Participants reported the proportion of people in five social groups (immediate family, extended family, friends and acquaintances, people at work/school, healthcare professionals) who were aware they were LGBTQI using a five-point Likert scale. An overall outness scale was computed by taking the average of items (range 1-5) with higher scores indicating participants were out to more people. The adapted measure had excellent internal consistency (Cronbach’s α=.902).

#### 2.3.9 Social support

The social support subscale of the Health Literacy Questionnaire (99) was used to assess social support. Participants were asked to rate their agreement with five statements on whether they were supported by others, using a five-point Likert scale (*strongly agree* to *strongly disagree*). Items included access to several people for support, feeling understood by others, having a person to attend medical appointments with, and strength of support. An overall social support score was computed by taking the average of items (range 1-5, higher scores indicating stronger support). In this study, the scale had good internal consistency (Cronbach’s α=.842). Participants were also asked who their primary support people were during the cancer experience.

### 2.4 Data handling and analysis

#### 2.4.1 Data cleaning

All survey responses were downloaded from Qualtrics into IBM’s Statistical Package for the Social Sciences (SPSS). Participant responses were screened and excluded if they had not completed any survey measures beyond demographics/cancer characteristics (n=630), were not LGBTQI (n=6), or had only entered non-serious or nonsensical responses (n=2). Thirteen cases were identified where participants had completed the survey multiple times, as identified through IP addresses, provided contact details, and responses. In these instances, the more complete survey was retained (or the earliest recorded, where completion was the same across records). The final dataset comprised 430 surveys.

#### 2.4.2.Statistical analyses

##### 2.4.2.1 Comparing psycho-social variables across LGBTQI groupings

Sexuality and gender identity questions were developed and recoded following advice from our LGBTQI partner investigators and stakeholder group. Gender was recoded into three categories (cis female, cis male, and TGD), based on participants’ self-reported gender (male, female, non-binary, other) and sex assigned at birth. Sexuality was recoded into three categories, lesbian/gay/homosexual, bisexual, and queer. The variable capturing whether participants had intersex variations retained two categories (yes, no). Age at survey completion was converted into a categorical variable, with participants classified as adolescents and young adults (AYAs, 15-39 years) or older adults (40+ years), following published recommendations for definition of AYA status (100). Cancer types were categorized as reproductive (breast, gynecological, prostate, testicular) or non-reproductive cancers, following previous research (101).

Analyses of variance (ANOVAs) were run to explore differences in distress, QOL and psycho-social variables previously reported to be associated with distress and QOL in LGBTQI cancer populations (sexual and physical concerns, impacts on LGBTQI and gender identities, minority stress variables, fear of cancer recurrence, and social support), by gender, sexuality, intersex variation, age, cancer type, and geographical remoteness. A Bonferroni correction was applied to account for the increased potential for type I errors when running multiple comparisons. An alpha cut-off of.008 (.05 divided by 6 types of between-group testing) was used to indicate significance. Ten TGD and intersex participants who identified as heterosexual were excluded from analyses of differences between sexualities, due to small sample size. These participants were included in other analyses. All other participants were included in each ANOVA, based on the grouping demographic variable of interest. Valid percentages are presented in the reporting of results and the proportion of participants responding to each measure.

##### 2.4.2.2 Identifying factors associated with distress and QOL

Bivariate correlation analyses were conducted to examine the association between distress and QOL and factors potentially associated with distress and QOL (sexual and physical concerns, impacts on LGBTQI and gender identities, minority stress variables, fear of cancer recurrence, and social support). These analyses were run for the whole sample and for subgroups defined by gender, sexuality, intersex status, age, cancer type and geographical remoteness. Chi-square test was used to compare equality of independent correlation coefficients, standardized for analysis, to assess differences in observed correlations for distress and QOL by gender, sexuality, intersex status, age, cancer type, and geographical remoteness.

## 3 Results

### 3.1 Participant characteristics

**Tables 1**, **2** present the demographic and cancer characteristics of survey respondents, respectively. Most participants were cisgender (83.9%; 50.2% cis women, 33.7% cis men), Caucasian (85.2%) older adults (77.9%), living in Australia (72.3%), who identified themselves as lesbian, gay, or homosexual (73.7%). Greater diversity was evident in participants’ geographical regional (54.4% urban; 33.8% regional; 11.7% rural or remote), and cancer types **(** **Table 2**). A minority of participants identified as TGD (14.7%), bisexual (10.9%), or queer (10.5%); 7.2% reported an intersex variation. A minority identified as Australian Aboriginal, Torres Strait Islander or Maori (2.1%), Asian (2.6%), or from a mixed ethnic background (4.5%). A range of cancer types were represented, including both reproductive (32.4%) and non-reproductive (67.6%) cancers.

**Table 1:** Demographic characteristics of survey participants.

Demographic Characteristic	*N*	*M (SD) *range
Age at time of study (years)	429	52.5 (15.7), 16-92
	***N***	***n* (%)**
Country	430	
Australia United States of America United Kingdom New Zealand Canada Other		311 (72.3%) 62 (14.4%) 29 (6.7%) 8 (1.9%) 7 (1.6%) 13 (3.0%)
Location	429	
Urban Regional Rural or remote		234 (54.5%) 145 (33.8%) 50 (11.7%)
Race/ethnicity	425	
Caucasian Asian Australian Aboriginal, Torres Strait Islander or Maori Mixed background Other/unclear background		362 (85.2%) 11 (2.6%) 9 (2.1%) 19 (4.5%) 24 (5.6%)
Gender	430	
Cis female Cis male TGD^1^ Different identity		216 (50.2%) 145 (33.7%) 63 (14.7%) 6 (1.4%)
Sexuality	430	
Lesbian, gay or homosexual Bisexual Queer Straight or heterosexual Different or multiple identities		317 (73.7%) 47 (10.9%) 45 (10.5%) 10 (2.3%) 11 (2.6%)
Intersex variation	430	
Yes No Prefer not to answer		31 (7.2%) 388 (90.2%) 11 (2.6%)
Relationship status^2^	368	
Not in a relationship Casually dating Relationship with one other person Multiple relationships		126 (34.2%) 16 (4.3%) 216 (58.7%) 16 (4.3%)
Social support network	374	
Partner/s Parents Other family Friends Colleagues Other No support people		226 (60.4%) 94 (25.1%) 130 (34.8%) 189 (50.5%) 46 (12.3%) 14 (3.7%) 35 (9.4%)
Supported by other LGBTQI people	418	318 (76.1%)
Education	422	
Less than secondary Secondary Some post-secondary Post-secondary		10 (2.4%) 45 (10.7%) 55 (13.0%) 312 (73.9%)

^1^34 (7.9%) non-binary, 13 (3.0%) trans female, 8 (1.9%) trans male, 8 (1.9%) different TGD identity; ^2^Participants could indicate multiple options if applicable

**Table 2:** Cancer characteristics of survey participants.

Cancer Characteristic	*N*	*M (SD), *range
Age at diagnosis (years)	363	46.3 (15.3), 1-79
	***N***	***n* (%)**
Medical intervention for cancer risk	430	74 (17.2%)
Cancer diagnosis (first)	370	
Brain Breast Cervical Colorectal Head/neck Leukaemia Lymphoma Ovarian Prostate Skin Uterine Other Not sure or unknown		11 (3.0%) 90 (24.3%) 11 (3.0%) 17 (4.6%) 14 (3.8%) 17 (4.6%) 24 (6.5%) 17 (4.6%) 59 (15.9%) 25 (6.8%) 23 (6.2%) 58 (15.7%) 4 (1.1%)
Cancer stage	369	
Localised Regional Distant/metastatic N/A (e.g. blood cancer) Not sure or unclear		228 (61.8%) 88 (23.8%) 32 (8.7%) 5 (1.4%) 16 (4.3%)
Treatment status	370	
No treatment yet On active curative treatment On maintenance treatment In remission Receiving palliative care (no further active treatment) Not sure		37 (10.0%) 37 (10.0%) 60 (16.2%) 217 (58.6%) 4 (1.1%) 8 (2.2%)
Subsequent cancers^1^	370	
Recurrence New primary cancer		57 (15.4%) 40 (10.8%)
Other health condition, disability or impairment	338	135 (39.9%)

^1^Participants could indicate multiple options if applicable.

### 3.2 Distress and QOL

Addressing research question 1, means and standard deviations for distress and QOL for the whole sample and by gender, sexuality, intersex variation, age, and cancer type, are reported in **Table 3**. Of 316 participants who completed the K10, 114 (36.1%) reported low distress, 73 (23.1%) reported moderate distress, 73 (23.1%) reported high distress, and 56 (17.7%) reported very high distress. The mean distress score for the sample was 20.9 (*SD =* 8.6, range 10-48), and the mean QOL score was 4.7 (*SD* = 1.6, range 1-7).

**Table 3:** Means and standard deviations of distress and quality of life, for total sample and subgroups.

Variable	Total sample*M(SD)*	Gender			Sexuality			Intersex status		Age		Remoteness*			Cancer type	
		Cis women*M(SD)*	Cis men*M(SD)*	TGD*M(SD)*	Lesbian/gay*M(SD)*	Bisexual*M(SD)*	Queer*M(SD)*	Intersex*M(SD)*	Non-intersex*M(SD)*	AYA*M(SD)*	Older adult*M(SD)*	Urban*M(SD)*	Regional*M(SD)*	Rural*M(SD)*	Reprod *M(SD)*	Non-reprod *M(SD)*
Distress	20.9 (8.6)	20.0 (7.6)	20.4 (8.9)	**25.6 (10.6)**	19.7 (8.1)	**24.9 (9.1)**	23.3 (8.7)	25.3 (8.4)	20.3 (8.4)	**25.9 (9.3)**	19.5 (7.9)	19.5 (8.6)	**22.7 (8.4)**	**22.7 (8.1)**	19.2 (8.7)	21.0 (8.3)
QOL	4.7(1.6)	**4.9 (1.4)**	4.8 (1.5)	3.7 (1.8)	**5.0 (1.4)**	4.2 (1.5)	3.9 (1.5)	3.6 (1.6)	**4.8 (1.5)**	4.3 (1.6)	4.8 (1.5)	4.8 (1.5)	4.5 (1.6)	4.8 (1.7)	5.0 (1.5)	4.8 (1.5)

Where differences between groups are statistically significant (p≤.008, using Bonferroni correction), the highest value/s are bolded. *Urban, in a major city of 100,000+ people, or the surrounding suburbs; Regional, in a smaller city; Rural, outside of a city. AYA, adolescent and young adult (15-39 years); cis, cisgender; QOL, quality of life; reprod, reproductive; TGD, transgender and gender diverse.

Distress differed significantly by gender, sexuality, age and geographical remoteness: higher distress was reported by TGD participants, relative to cis men and women (*F_2,309 =_* 7.084, *p*=.001); by bisexual and queer participants, relative to lesbian/gay participants (*F_2,302 =_* 8.095, *p*<.001); by AYAs, relative to older adults (*F_1,314 =_* 31.959, *p*<.001); and by those living in rural or regional areas compared to those living in urban areas (*F_2,313 _* =5.557, *p*<.004). Distress did not differ significantly between those with and without intersex variations after Bonferroni correction; or between reproductive and non-reproductive cancers (see **Appendix Table A1** for effect sizes and statistics). QOL also varied significantly by gender, sexuality and intersex status: higher QOL was reported by cis women and men, relative to TGD participants (*F_2,326 =_* 12.167, *p*<.001); by lesbian/gay participants, relative to bisexual and queer participants (*F_2,318 =_* 12.718, *p*<.001); and by those without intersex variations, relative to those with intersex variations (*F_1,324 =_* 16.360, *p*<.001). QOL did not differ significantly by age (after Bonferroni correction), cancer type or geographical remoteness (**Appendix Table A1**).

### 3.3 Comparing psycho-social variables associated with distress and QOL between LGBTQI groups

Addressing research question 2, **Table 4** presents the means and standard deviations of study variables (sexual and physical concerns, impacts on LGBTQI and gender identities, minority stress variables, fear of cancer recurrence, and social support), for the whole sample, and for subgroups defined by gender identity, sexuality, intersex status, age and cancer type. Statistics relating to the tests of differences are presented in **Appendix Table A2** and summarized in the text where significant differences were found.

**Table 4:** Means and standard deviations of study variables, for total sample and subgroups.

Variable	Total sample*M(SD)*	Gender			Sexuality			Intersex status		Age		Remoteness*			Cancer type	
		Cis women*M(SD)*	Cis men*M(SD)*	TGD*M(SD)*	Lesbian/gay*M(SD)*	Bisexual*M(SD)*	Queer*M(SD)*	Intersex*M(SD)*	Non-intersex*M(SD)*	AYA*M(SD)*	Older adult*M(SD)*	Urban*M(SD)*	Regional*M(SD)*	Rural*M(SD)*	Reprod *M(SD)*	Non-reprod *M(SD)*
Sexual concerns	3.6 (3.2)	3.5 (3.1)	4.0 (3.2)	2.9 (3.1)	3.6 (3.2)	4.0 (3.1)	3.9 (3.2)	1.6 (2.4)	**3.7 (3.2)**	3.5 (3.1)	3.6 (3.2)	3.7 (3.3)	3.5 (3.0)	3.6 (3.3)	4.4 (3.2)	3.4 (3.0)
Physical concerns	5.3 (2.9)	5.6 (3.0)	4.8 (2.7)	5.6 (2.8)	5.1 (2.9)	5.6 (2.5)	**7.2 (2.1)**	5.3 (3.3)	5.3 (2.9)	**6.3 (2.9)**	5.0 (2.9)	5.1 (2.8)	5.7 (3.0)	5.2 (3.1)	5.1 (2.9)	5.5 (2.9)
LGBTQI impact	6.2 (2.4)	5.7 (2.2)	6.5 (2.5)	**7.1 (2.7)**	6.1 (2.4)	6.1 (2.2)	6.8 (2.5)	6.5 (2.4)	6.1 (2.4)	6.6 (2.4)	6.1 (2.4)	6.1 (2.5)	6.3 (2.4)	6.1 (2.3)	6.4 (2.6)	6.1 (2.4)
Gender impact	1.6 (1.0)	1.4 (0.8)	1.4 (0.9)	**2.5 (1.3)**	1.4 (0.8)	1.9 (1.0)	**2.3 (1.3)**	2.1 (1.1)	1.5 (1.0)	**1.9 (1.2)**	1.5 (0.9)	1.6 (1.0)	1.6 (1.0)	1.6 (1.0)	1.6 (1.0)	1.5 (0.9)
FCR	2.4 (1.3)	2.3 (1.2)	2.3 (1.3)	2.5 (1.4)	2.3(1.3)	2.3(1.3)	2.7 (1.3)	2.1 (1.3)	2.4 (1.3)	2.7 (1.3)	2.3 (1.2)	2.4 (1.3)	2.3 (1.2)	2.5 (1.3)	2.3 (1.3)	2.4 (1.2)
Minority stress																
Discr (*gen*)	2.3 (0.9)	2.2 (0.8)	2.1 (0.9)	**2.8 (1.0)**	2.2 (0.8)	2.3 (0.9)	2.5 (0.9)	**2.8 (1.0)**	2.2 (0.8)	**2.5 (0.9)**	2.2 (0.8)	2.2 (0.9)	2.3 (0.9)	2.3 (0.9)	2.1 (0.8)	2.2 (0.8)
Discr (*care*)	1.5 (0.8)	1.4 (0.6)	1.4 (0.8)	**2.0 (1.1)**	1.4 (0.8)	1.3 (0.6)	**1.9 (1.1)**	**2.2 (1.2)**	1.4 (0.7)	**1.7 (0.9)**	1.4 (0.8)	1.5 (0.8)	1.5 (0.8)	1.6 (0.9)	1.4 (0.8)	1.4 (0.7)
Discomf LGBTQI	5.8 (2.5)	5.6 (2.4)	5.6 (2.3)	**7.1 (2.8)**	5.5 (2.2)	**7.6 (3.0)**	6.1 (2.6)	**7.0 (2.9)**	5.7 (2.4)	**7.0 (2.8)**	5.5 (2.3)	5.8 (2.4)	5.9 (2.5)	6.0 (2.8)	5.5 (2.2)	5.9 (2.5)
Outness	4.1 (1.1)	4.1 (1.1)	**4.3 (0.9)**	3.7 (1.1)	**4.4 (0.8)**	2.8 (1.1)	3.9 (1.0)	3.8 (1.0)	4.2 (1.0)	3.5 (1.1)	**4.3 (1.0)**	4.2 (1.0)	4.0 (1.1)	4.1 (1.1)	4.2 (1.0)	4.1 (1.0)
Social support	3.9 (0.9)	4.1 (1.1)	**4.3 (0.9)**	3.7 (1.1)	**4.0 (0.9)**	3.5 (1.0)	3.8 (0.9)	3.4 (0.9)	**3.9 (0.9)**	3.6 (0.8)	**4.0 (0.9)**	3.9 (0.9)	3.8 (1.0)	3.9 (0.8)	4.0 (0.8)	4.0 (0.9)

Where differences between groups are statistically significant (p≤.008, using Bonferroni correction), the highest value/s are bolded. *Urban, in a major city of 100,000+ people, or the surrounding suburbs; Regional, in a smaller city; Rural, outside of a city. AYA, adolescent and young adult (15-39 years); cis, cisgender; discomf LGBTQI, discomfort being LGBTQI; discr (care), discrimination in cancer care; discr (gen), discrimination in general life; FCR, fear of cancer recurrence; QOL, quality of life; reprod, reproductive; TGD, transgender and gender diverse.

#### 3.3.1 Sexual concerns

Concerns about changes to sexual wellbeing since cancer were reported by 71.3% (n=275) of participants, with these participants reporting 3.60 concerns on average (*SD* = 3.18, range 0-10). Participants who indicated that the question was not applicable, because they were diagnosed or had medical intervention for cancer as children, were excluded from the analysis. The most commonly endorsed sexual concerns were decreased satisfaction with the level of sexual desire (48.5%), decreased satisfaction with sex life (43.8%), fatigue or lack of energy affecting sex life (43.1%), decreased satisfaction with the ability to orgasm (39.9%), decreased enjoyment of sexual activity (39.2%) and decreased satisfaction with physical intimacy (37.5%).

Participants with intersex variations reported significantly lower sexual concerns than participants without intersex variations (*F_1,265 =_* 7.433, *p*=.007). There were no significant differences in sexual concerns by gender, sexuality, age, cancer type (after Bonferroni correction) or geographical remoteness.

#### 3.3.2 Physical concerns

Participants reported 5.3 physical concerns on average (*SD* = 2.9, range 0-12, N=303). The physical concerns reported included reduced body strength (69.0%), muscle loss/wastage (61.5%), weight gain (58.2%), reduced mobility (55.4%), scarring (52.0%), changes in genital sensitivity (45.7%), incontinence (40.9%), hair loss (37.5%), early menopause (30.8%) and weight loss (25.3%); loss of one/both breasts 54 (16.7%); shortened penis 54 (16.7%); stoma 16 (5.0%).

Significantly higher physical concerns were reported by AYAs compared to older adults (*F_1,301 =_* 10.235, *p*=.002), and by participants who identified as queer, compared to those who identified as lesbian, gay or bisexual (*F_2,291 =_* 7.993, *p*<.001). There were no significant differences in physical concerns by gender, intersex status, cancer type, or geographical remoteness.

#### 3.3.3 Impact of cancer on LGBTQI identity and gender identity

Many participants reported that their cancer and cancer care had impacted upon their experiences as LGBTQI people. Overall, 173 (41.3%) participants reported cancer impact on their feelings about being LGBTQI (LGBQ n=147, 37.5%, TGD n=25, 59.5%, intersex n=15, 69.2%). 280 (66.7%) reported impact of cancer on openness about being LGBTQI (LGBQ n=244, 65.6%, TGD n=31, 73.8%, intersex n=14, 60.9%). Impact in involvement with LGBTQI communities was reported by 250 (59.4%) participants (LGBQ n=216, 57.9%, TGD n=30, 71.4%, intersex n=12, 52.2%). Additionally, 101 (30.5%) participants reported that cancer had impacted upon their feelings about their gender identity, as a man, woman, transgender, non-binary or gender fluid person.

Impact on LGBTQI identity was significantly higher for TGD participants than cis women and cis men (*F_2,408 =_* 9.308, *p*<.001). There were no significant differences in impact on LGBTQI identity by sexuality, intersex status, age, cancer type or geographical remoteness.

Impact on gender identity was significantly higher for TGD participants than cis women and cis men (*F_2,323 =_* 27.245, *p*<.001); for queer participants in comparison to those who identified as gay/lesbian or bisexual (*F_2,316 =_* 21.586, *p*<.001); and for AYAs compared to older adults (*F_1,329 =_* 9.535, *p*<.002). There were no significant differences in cancer impact on gender identity by intersex status (after Bonferroni correction), cancer type or geographical remoteness.

#### 3.3.4 Fear of cancer recurrence

Two-thirds of participants (67.0%) reported that they were afraid of their cancer recurring. There were no significant differences in FCR by gender, sexuality, intersex status, age (after Bonferroni correction), cancer type or geographical remoteness.

#### 3.3.5 Minority stress

##### 3.3.5.1 Discrimination in general life and cancer care

**Figure 1:**
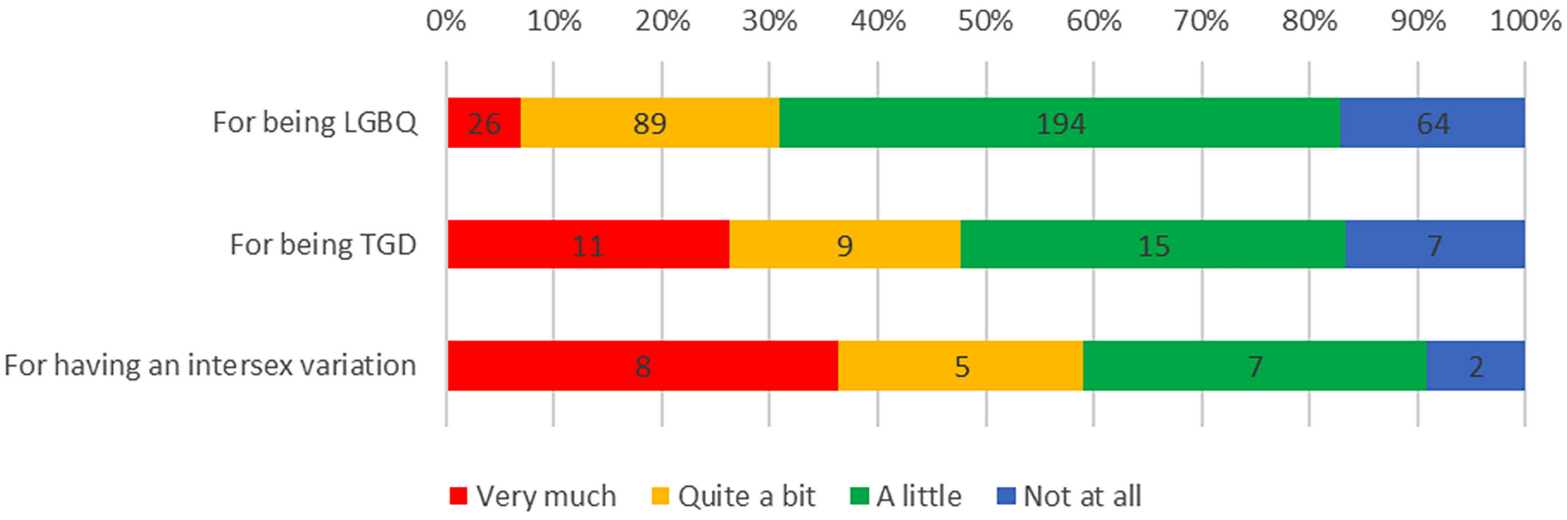
Experiences of Discrimination for being LGBQ, TGD, or for having an Intersex Variation.

**Figure 2:**
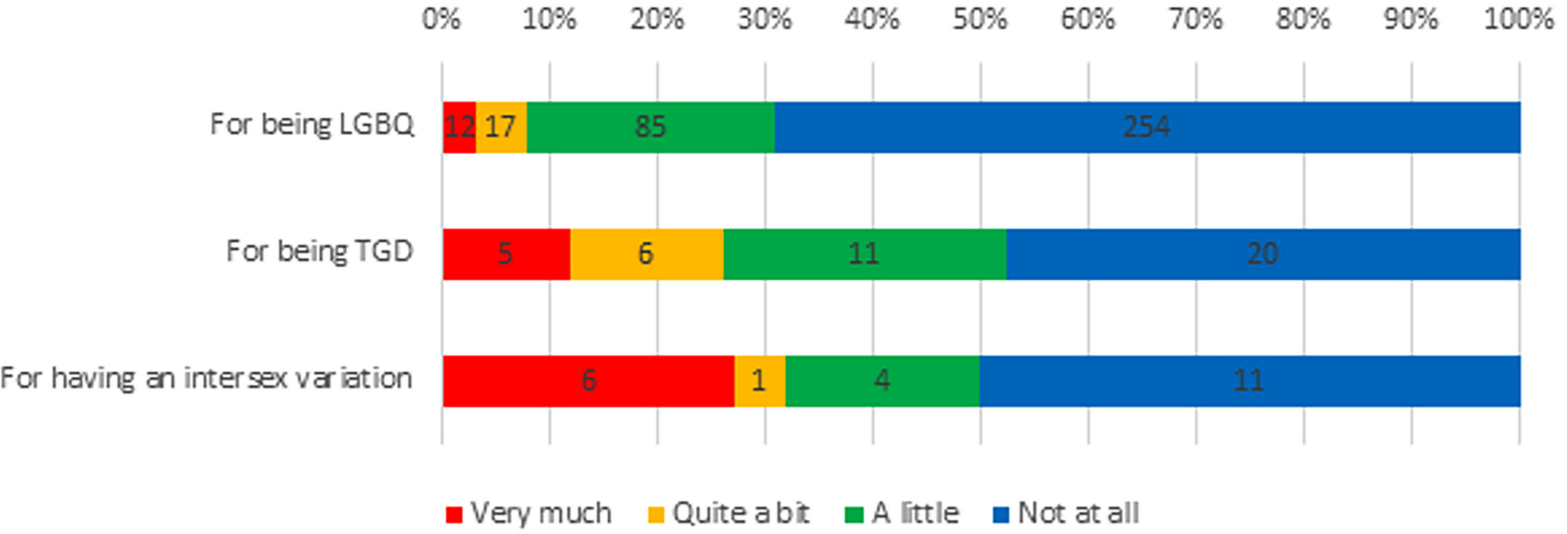
LGBTQI+ Experiences of Discrimination in Cancer Care. LGBQ, lesbian, gay, bisexual or queer; TGD, transgender/gender diverse.

Experiences of discrimination were common among respondents: 351 (83.6%) reported discrimination in their life in general, including 309 (82.8%) LGBQ participants, 35 (83.3%) TGD participants and 20 (90.9%) participants born with intersex variations, because of their sexuality, TGD status, or intersex variation, respectively (**Figure 1**). Furthermore, a third of participants (n=138, 33%) reported experiencing discrimination as part of their cancer care because of being LGBTQI, including 104 (31.0%) LGBQ participants, 22 (52.4%) TGD participants and 11 (50.0%) participants with intersex variations (**Figure 2**).

Significantly higher discrimination in life was reported by TGD participants compared to cisgender women and men (*F_2,411 =_* 13.476, *p*<.001); by intersex compared to non-intersex participants (*F_1,408 =_* 13.556, *p*<.001); and by AYAs compared to older adults (*F_1,417 =_* 7.876, *p*=.005). There were no differences in reporting of discrimination in life by sexuality, cancer type or geographical remoteness.

Significantly higher discrimination in cancer care was reported by TGD participants compared to cisgender women and men (*F_2,406 =_* 15.886, *p*<.001); by queer participants in comparison to gay/lesbian and bisexual participants (*F_2,397 =_* 6.556, *p*=.002); by intersex compared to non-intersex participants (*F_1,403 =_* 27.439, *p*<.001); and by AYAs compared to older adults (*F_1,412 =_* 7.413, *p*<.007). There were no differences in reporting of discrimination in cancer care between participants with reproductive and non-reproductive cancers, or by geographical remoteness.

##### 3.3.5.2 Discomfort in being LGBTQI

Most participants agreed that they were comfortable being LGBTQI (n=383; 91.0%), with greater comfort reported by LGBQ participants (n=347, 93.0%) compared to TGD (n=35, 83.3%) and intersex participants (n=16, 69.6%). A small proportion of the sample (n=29, 6.9%) wished they were not LGBTQI, reflecting relatively low levels of internalized prejudice, including 20 (5.4%) LGBQ participants, 8 (19.0%) TGD participants and 4 (17.4%) intersex participants. A large proportion of participants kept careful control over who knew they were LGBTQI (n=128, 30.8%), reflecting concealment motivation: 104 (28.3%) LGBQ participants, 23 (54.8%) TGD participants and 12 (52.2%) participants with intersex variations.

Significantly greater discomfort in being LGBTQI was reported by TGD participants compared to cisgender women and men (*F_2,403 =_* 13.476, *p*<.001); by bisexual compared to gay/lesbian and queer participants (*F_2,394 =_* 17.493, *p*<.001); by intersex compared to non-intersex participants (*F_1,401 =_* 13.556, *p*<.001); and by AYAs compared to older adults (*F_1,409 =_* 24.698, *p*<.001). There were no differences in discomfort in being LGBTQI by cancer type or by geographical remoteness.

##### 3.3.5.3 Outness

The average score on the outness measure was 4.09 (*SD* = 1.08). On average, participants were most likely to have disclosed that they were LGBTQI to immediate family (*M* = 4.41, *SD* = 1.18) and friends/acquaintances (*M* = 4.37, *SD* = 0.90), followed by general HCPs (*M* = 4.06, *SD* = 1.35), extended family (*M* = 3.90, *SD* = 1.39) and at work/school (*M* = 3.84, *SD* = 1.31).

Cisgender men were significantly more likely to be out compared to cisgender women and TGD participants (*F_2,393 =_* 7.448, *p*<.001); significantly more gay/lesbian participants were out, compared to bisexual and queer participants (*F_2,385 =_* 54.461, *p*<.001); and older adults were more likely to be out than AYAs (*F_1,399 =_* 39.800 *p*<.001). There were no differences in outness by intersex status, cancer type, or geographical remoteness.

#### 3.3.6 Social support

Current social support was generally high amongst participants, with the majority agreeing that they had strong support from family and friends (n=289, 78.3%), could get access to several people who understand and support them (n=296, 79.5%) and had at least one person who could attend medical appointments with them (n=291, 79.8%). The mean social support score was 3.88 (*SD* 0.92, range 1-5). When asked to report their primary support people during their cancer experience, participants largely nominated intimate partners (n=226, 60.4%), friends (n=189, 50.5%), parents (n=94, 25.1%), other family (n=130, 34.8%), and colleagues (n=46, 12.3%). A minority (n=35, 9.4%) reported that they did not have support people at the time. Most participants (n=232, 63.0%) had one intimate partner (n=216, 58.7%), with a minority having multiple partners (n=16, 4.3%)^1^.

Social support was significantly higher for cisgender men compared to cisgender women and TGD participants (*F_2,365 =_* 7.448, *p*<.001); higher for gay/lesbian compared to bisexual and queer participants (*F_2,357 =_* 6.577, *p*=.002); higher for non-intersex compared to intersex participants (*F_1,363 =_* 9.338, *p*=.002); and higher for older adults compared to AYAs (*F_1,372 =_* 9.585, *p*=.002). There were no differences in social support by cancer type or geographical remoteness.

### 3.4 Identifying psycho-social variables associated with distress and QOL

Addressing research question (RQ) 3 and 4, **Tables 5**, **6** presents the analysis examining bivariate correlations between potential predictors of distress and QOL, for the sample as a whole and for subgroups, comparing by gender, sexuality, intersex status, age and cancer type. Tables **Appendix Table A3** and **Appendix Table A4** report differences in the correlations within subgroups. In the whole sample (RQ 3), distress was significantly positively correlated with discomfort with being LGBTQI, discrimination in general life and in cancer care, physical and sexual concerns, and impact on LGBTQI and gender identity. Distress was negatively correlated with QOL, outness, and social support. Additionally, QOL was positively correlated with outness and social support, and negatively correlated with discomfort with being LGBTQI, discrimination in life and cancer care, physical concerns, and impact on LGBTQI and gender identity.

**Table 5:** Correlations between distress and other study variables, for total sample and subgroups.

Variable	Total sample	Gender			Sexuality			Intersex status		Age		Remoteness*			Cancer type	
		Cis women	Cis men	TGD	Lesbian/gay	Bisexual	Queer	Intersex	Non-intersex	AYA	Older adult	Urban	Regional	Rural	Reprod	Non-reprod
QOL	-.606^**^	-.564^**^	-.605^**^	-.578^**^	-.591^**^	-.438^**^	-.509^**^	-.315	-.622^**^	-.561^**^	-.613^**^	-.588^**^	-.629^**^	-.633^**^	-.755^**^	-.541^**^
Sexual concerns	.204^**^	.321^**^	.189	.044	.250^**^	.364^**^	-.091	.319	.221^**^	.235	.211^**^	.206^**^	.231^**^	.158	.225	.152
Physical concerns	.356^**^	.359^**^	.379^**^	.360^**^	.364^**^	.394^**^	.046	.182	.356^**^	.263^**^	.337^**^	.385^**^	.307^**^	.288	.488^**^	.323^**^
LGBTQI impact	.210^**^	.223^**^	.144	.163	.258^**^	-.077	.163	.079	.186^**^	.295^**^	.155^**^	.229^**^	.172	.220	.168	.242^**^
Gender impact	.258^**^	.330^**^	-.066	.089	.266^**^	.057	.172	.409	.219^**^	.253	.208^**^	.203^**^	.312^**^	.339^**^	.316^**^	.214^**^
FCR	.448^**^	.345^**^	.383^**^	.403^**^	.414^**^	.461^**^	.143	.120	.384^**^	.143	.417^**^	.456^**^	.210^**^	.381^**^	.451^**^	.316^**^
Minority stress																
Discr (*gen*)	.265^**^	.287^**^	.130	.222	.281^**^	.288	-.156	.222	.195^**^	.062	.307^**^	.230^**^	.241^**^	.487^**^	.124	.388^**^
Discr (*care*)	.271^**^	.253^**^	.220^**^	.156	.275^**^	.177	-.002	-.135	.249^**^	.006	.339^**^	.294^**^	.164	.416^**^	.261^**^	.267^**^
Discomf LGBTQI	.309^**^	.282^**^	.326^**^	.222	.261^**^	.204	.380^**^	.575^**^	.269^**^	.279^**^	.231^**^	.308^**^	.336^**^	.285	.239^**^	.374^**^
Outness	-.216^**^	-.297^**^	-.106	-.002	-.147^**^	-.267	.027	-.256	-.196^**^	-.185	-.116	-.168^**^	-.285^**^	-.166	-.127	-.265^**^
Social support	-.475^**^	-.535^**^	-.403^**^	-.342^**^	-.425^**^	-.662^**^	-.627^**^	-.314	-.475^**^	-.542^**^	-.428^**^	-.518^**^	-.415^**^	-.448^**^	-.445^**^	-.489^**^
Social support	.417^**^	.455^**^	.391^**^	.199	.436^**^	.394^**^	.222	-.314	.442^**^	.409^**^	.406^**^	.365^**^	.454^**^	.547^**^	.336^**^	.471^**^

*Urban, in a major city of 100,000+ people, or the surrounding suburbs; Regional, in a smaller city; Rural, outside of a city. **p≤.05; significant correlations indicated in bold. AYA, adolescent and young adult (15-39 years); cis, cisgender; discomf LGBTQI, discomfort being LGBTQI; discr (care), discrimination in cancer care; discr (gen), discrimination in general life; FCR, fear of cancer recurrence; QOL, quality of life; reprod, reproductive; TGD, transgender and gender diverse.

**Table 6:** Correlations between QOL and other study variables, for total sample and subgroups.

Variable	Total sample	Gender			Sexuality			Intersex status		Age		Remoteness*			Cancer type	
		Cis women	Cis men	TGD	Lesbian/gay	Bisexual	Queer	Intersex	Non-intersex	AYA	Older adult	Urban	Regional	Rural	Reprod	Non-reprod
Sexual concerns	-.097	-.186^**^	-.095	.032	-.162^**^	-.372^**^	.368	.039	-.139^**^	-.114	-.096	-.106	-.003	-.265	-.199	-.098
Physical concerns	-.300^**^	-.296^**^	-.327^**^	-.289	-.300^**^	-.295	-.151	.121	-.337^**^	-.284^**^	-.284^**^	-.303^**^	-.220^**^	-.435^**^	-.399^**^	-.297^**^
LGBTQI+ impact	-.149^**^	-.150^**^	-.124	.064	-.146^**^	-.293	-.072	.081	-.138^**^	-.313^**^	-.093	-.127	-.213^**^	-.067	-.242^**^	-.147^**^
Gender impact	-.281^**^	-.229^**^	-.287^**^	.060	-.208^**^	-.145	-.153	-.198	-.244^**^	-.317^**^	-.251^**^	-.195^**^	-.443^**^	-.224	-.398^**^	-.197^**^
FCR	-.190^**^	-.123	-.236^**^	-.253	-.190^**^	-.215	-.267	-.037	-.217^**^	-.196	-.173^**^	-.332^**^	.036	-.141	-.222^**^	-.213^**^
Minority stress																
Discr (*gen*)	-.193^**^	-.193^**^	.001	-.203	-.199^**^	.127	.054	-.164	-.130^**^	-.168	-.186^**^	-.096	-.318^**^	-.312^**^	-.041	-.251^**^
Discr (*care*)	-.226^**^	-.172^**^	-.094	-.230	-.145^**^	-.003	-.295	.231	-.198^**^	-.310^**^	-.183^**^	-.258^**^	-.178	-.223	-.248^**^	-.183^**^
Discomf LGBTQI	-.240^**^	-.193^**^	-.287^**^	-.126	-.193^**^	-.238	-.080	-.206	-.212^**^	-.156	-.237^**^	-.227^**^	-.201^**^	-.374^**^	-.198	-.282^**^
Outness	.222^**^	.196^**^	.216^**^	.110	.195^**^	.339^**^	-.217	.176	.209^**^	.225	.183^**^	.186^**^	.228^**^	.299	.184	.253^**^
Social support	.417^**^	.455^**^	.391^**^	.199	.436^**^	.394^**^	.222	-.314	.442^**^	.409^**^	.406^**^	.365^**^	.454^**^	.547^**^	.336^**^	.471^**^

*Urban, in a major city of 100,000+ people, or the surrounding suburbs; Regional, in a smaller city; Rural, outside of a city. **p≤.05; significant correlations indicated in bold. AYA, adolescent and young adult (15-39 years); cis, cisgender; discomf LGBTQI, discomfort being LGBTQI; discr (care), discrimination in cancer care; discr (gen), discrimination in general life; FCR, fear of cancer recurrence; QOL, quality of life; reprod, reproductive; TGD, transgender and gender diverse.

For most subgroups (RQ 4), physical concerns, FCR, discomfort in being LGBTQI, and social support were significantly associated with distress and QOL, in the same direction as for the whole sample. For some of the subgroups with relatively small participant numbers (TGD, bisexual, queer, intersex, AYA), several the correlations failed to reach significance, suggesting larger sample size may reach significance. These findings suggest that higher physical concerns, higher FCR, greater discomfort in being LGBTQI, and lower social support are associated with higher distress and lower QOL for most participants, when compared across subgroups. There were few significant differences within subgroups in correlations (Table A3 and A4). The association between distress and impact of cancer on gender identity varied significantly by gender and was higher for cisgender women than for cisgender men and TGD participants (A3). Associations between social support and QOL were more positive in non-intersex participants, but did not reach significance for intersex participants (A4).

## 4 Discussion

This is the first large scale study to systematically examine distress and QOL and key psycho-social concomitants for LGBTQI people with cancer, comparing intersecting identity groups, including cisgender and TGD, intersex and non-intersex, lesbian/gay, bisexual and queer, AYAs and older adults, reproductive and non-reproductive tumor types, and those living in urban, rural and regional areas.

Average levels of distress for the whole sample were comparable or slightly elevated relative to a recent Australian study of predominantly heterosexual cisgender cancer survivors (101) and Australian cancer population reference values using the same measure (102). Similarly, the average QOL rating was almost identical to EORTC cancer population reference data (90). However, the proportion of participants reporting high or very high distress levels in the present study (41%) was approximately three to six times higher than previous Australian cancer population studies using the same measure (7-12%) (103–105). This finding confirms previous reports of greater distress in LGBTQI cancer populations, in comparison with non-LGBTQI cancer populations (1, 2). Levels of high distress were also proportionately greater than rates of depression and anxiety reported in previous cancer research with LGB people. For example, a study of sexual minority breast cancer survivors (43) recorded clinically relevant depression and anxiety in 31% and 25% of participants respectively. A study of LGB people with gynecological cancer (17) reported depression and anxiety in 32% at 25% of participants respectively; and clinical levels of distress were reported by 13.7% of participants in a study of gay and bisexual men with prostate cancer (9).

The higher rates of distress reported in the present study in comparison with previous LGBTQI cancer research can be interpreted in relation to variations identified in intersecting identity sub-groupings. Significantly higher levels of distress and lower QOL were found in TGD, AYA, queer and bisexual sub-groups, in comparison with cisgender, older, lesbian/gay sub-groups—the later sub-groups have been the focus of previous LGBTQI cancer research (1, 2). Rates of QOL were significantly lower in intersex compared to non-intersex groups, with rates of distress close to significance. In combination, this suggests that psychological outcomes may be worse for LGBTQI people with cancer than has previously been estimated (106) as there has been a dearth of research that included TGD, AYA and intersex people with cancer, as well as those who identified as bisexual or queer (1). These differences in health outcomes in LGBTQI sub-groups are reflected in differences in the psycho-social concomitants of distress and QOL, which can be conceptualized as intersecting stigma-related stressors (107). TGD, intersex, AYA, queer and bisexual subgroups reported higher levels of a number of these stressors, including discomfort with being LGBTQI, discrimination in life and in cancer care, lower outness, greater impact of cancer on LGBTQI identity and gender identity, and lower social support, likely contributing to their higher distress and poorer QOL.

These findings confirm previous reports of higher levels of societal discrimination (108) and discrimination in health care (109) reported by TGD people compared with other SGM groups. This is an explanation for higher rates of distress found in TGD populations outside of the context of cancer (56, 110), and impacts upon experiences of cancer survivorship and interactions with health care professionals (26, 60). In previous research, TGD people of color, and those who identify as LBQ, are at highest risk of discrimination, harassment and violence (111, 112). Individuals who have intersex variations also face societal discrimination and hostility (113), as well as normalizing medical interventions that are conducted in infancy without consent, serving to deny bodily integrity and autonomy (113, 114) and violate human rights (115). People with intersex variance experience a higher incidence of anxiety, depression and psychological distress compared with the general population, which has been linked to stigma and discrimination (116). Both TGD and people with an intersex variation continue to face pathologization in standardized psychiatric classification systems (115, 117), resulting in stigma and negative impact on identity and wellbeing (118). TGD and intersex individuals have been described as the most stigmatized and the least understood members of LGBTQI communities (116, 119). Prior to the present study, they were the least understood groups in LGBTQI cancer research (1, 2).

Previous research has noted that those who identify as queer (120) or bisexual (118, 121) report significantly higher rates of depression and anxiety when compared with people who identify as gay or lesbian (122). The findings of the present study confirm that this is the case with bisexual people with cancer, in line with a recent study that reported that bisexual women with cancer are more likely to report severe distress (12.5%) than lesbian (5.5%) and heterosexual (4.0%) women (16). This stands in contrast to other studies that have not reported differences in distress between bisexual and gay/lesbian people with cancer (43, 123). Higher rates of distress that have been observed in queer and bisexual individuals in the general LGBTQI population have been attributed to greater minority stress (120), associated with concealment of sexuality, struggles with identity and low social support (124). These findings are confirmed in the present study, in the context of queer and bisexual people with cancer, who report higher discomfort with being LGBTQI and greater impact on gender identity, with queer people reporting greater discrimination in cancer care, compared with lesbian/gay/homosexual identified participants. Indications that the direction of the association between some psycho-social variables and distress or QOL is different for the bisexual or queer sub-groups, in comparison to the lesbian/gay subgroup, although statistically non-significant, deserves further investigation. This includes impact of cancer on LGBTQI identity and discrimination in life in general, for the bisexual subgroup; sexual concerns, discrimination in general life and in cancer care, outness, for the queer subgroup.

AYAs are recognized to be a unique and complex population, reporting higher rates of distress and lower QOL than older adults with cancer (125). For example, a recent study reported that AYA cancer survivors report more anxiety (15.1% vs. 6.6%) and mood disorders (14.8% vs. 8.9%) than older adults (126). The only previous study of AYA SGM cancer survivors to date reported that cisgender women who identified as sexual minorities were twice as likely to experience anxiety than those who identified as heterosexual (19). The findings of the present study provide an explanation for this effect and demonstrate that AYA LGBTQI people with cancer are at higher risk of negative psycho-social outcomes than older adults (18). Adolescence and young adulthood is a time when many LGBTQI individuals define their sexual and gender identity, with increasing numbers of young people today estimated to be same sex attracted or gender diverse – 20-30% in recent Australian research (127). This can be a time when the effects and meanings of having a variation in sex characteristics are negotiated for the first time for intersex people (113). Whilst a cancer diagnosis interrupts any person’s developmental milestones, LGBTQI AYA survivors are vulnerable, because they risk rejection by family or friends when they “come out” or explore their gender identity (127), removing their main source of social support (128). Coming out can be a very difficult process for AYAs (129), reflected in the lower level of outness in AYAs in the present study. This is compounded for those who experience negative societal views or bullying (130), and by the double stigmatization of being an LGBTQI person with cancer (19, 131).

The higher rates of distress identified in rural and regional subgroups reinforces the need for attention to be made to the experiences and health care needs of LGBTQI people living outside of urban areas (77, 132). Higher rates of distress were not accompanied by higher levels of minority stress, or differences in any other psycho-social variables. This stands in contrast to previous research that identified higher minority stress and lower social support in LGBT people living in rural and regional Australia, in comparison to those living in urban areas in Australia (62). LGBT people living in rural areas of the USA also report high rates of minority stress (77) and difficulties in interactions with health care providers (132). In the qualitative arm of the *Out with Cancer Study*, some participants living in a rural or regional area reported social isolation and social stigma (60), whereas others reported high levels of community and health care practitioner support due to living in a “rural, small-town area where everyone knows everyone” and which contributed to “being respected” (26). There needs to be further investigation of LGBTQI cancer survivorship and care outside of urban areas, in order to understand potential health disparities experienced by rural and urban LGBTQI cancer survivors.

The lack of significant differences across cancer types suggests that LGBTQI people with reproductive and non-reproductive cancers experience similar levels of distress, minority stressors, and LGBTQI and gender impacts. This runs counter to healthcare professionals’ assumptions that sexual orientation, gender identity and intersex variations are only relevant to reproductive cancers (26), indicating that tailored support resources for LGBTQI communities are relevant across diagnoses. Given that previous studies have predominantly focused on reproductive cancers (1, 3), this necessitates further research into how LGBTQI people are impacted by other cancer types in order to inform subsequent resource development.

Our findings clearly demonstrate that for the sample as a whole group, distress and poor QOL are associated with physical and sexual concerns, the impact of cancer on gender and LGBTQI identities, minority stress (including discrimination in life and in cancer care, discomfort with being LGBTQI and outness to others), and lack of social support.

The association between concerns about physical and sexual changes after cancer and distress, reflect previous findings in the general cancer population (31, 133). Rates of physical and sexual concerns following cancer treatment were comparable to non-LGBTQI cancer populations (92, 134), and did not significantly differ across gender, sexuality, age or cancer type. A near significant trend towards higher sexual concerns in participants who had reproductive cancers confirms previous research (68, 133, 135, 136), and is deserved of further investigation. The finding of significantly lower sexual concerns in the intersex subgroups may be explained by the fact that many intersex participants had undergone medical intervention to avoid cancer as infants, as described in our qualitative analysis (60), rather than cancer treatment as adults, thereby avoiding the impact of cancer treatment on sexual wellbeing (137).

Physical and sexual changes associated with cancer can impact upon LGBTQI identity (37, 40) and gender identity (31, 32, 92, 138), factors found to be associated with distress and QOL for many participants in the present study. This is because embodiment is central to gendered and sexual identities (139–141). Our finding of a greater impact of cancer on gender identity in the TGD subgroup compared to cis male and female subgroups needs further investigation. The measure used in the survey did not ascertain the direction of the impact on gender identity – whether it was positive or negative. Qualitative findings from the *Out with Cancer* study (142), and previous research on TGD cancer survivorship (143, 144), suggest that cancer treatment can facilitate gender affirmation for some TGD people, resulting in a positive impact on gender identity. Future research should use a more complex measure of impact of cancer on gender and LGBTQI identity, ascertaining direction and nature of any impact, for all LGBTQI subgroups, alongside in-depth qualitative examination of identity impact.

Fear of cancer recurrence (FCR) was associated with distress, as reported in previous research in the general cancer population (49). We also found a significant association between FCR and low QOL, contrary to a recent study of non-LGBTQI cancer survivors, where no such association was found (145). Whilst there was no evidence of significant differences in FCR across LGBTQI identities or cancer type, there was a near significant trend towards higher FCR in AYAs, as reported in previous research (49, 52). There is a need for further research on FCR and its concomitants in LGBTQI people with cancer, across age groups.

It is widely accepted that high rates of distress found in the general LGBTQI population (55–57), and reported in previous research with cisgender LGB cancer survivors (1), are associated with minority stress (53, 54), as found in the present study. Minority stress theory (61) explains the link between stigma-related distal stressors in a person’s environment, such as LGBTQI discrimination, social rejection, homophobia and transphobia, and health. Research drawing on this framework suggests that living in a hostile, discriminatory context can elicit internal, health-eroding proximal stress processes related to individuals’ minority status, including anxious expectations of rejection, identity concealment, and internalized stigma (107, 146). This is reflected in the chronic stress experienced by LGBTQI people, as the result of stigmatization and discrimination within heterosexist and transphobic societies (147, 148).

Minority stress is acute in contexts where, until recently, LGBTQI relationships did not have the same status as heterosexual relationships (55, 149). There is evidence of LGBTQI discrimination in Australia (150) and the USA (151), where the majority of our participants reside. This is manifested by political and public debate about the right of religious organizations, schools, and health practitioners to exclude or discriminate against LGBTQI people (150). Homophobic and transphobic public discourse associated with marriage equality debates have been described as an act of “symbolic violence” (149). For young LGBTQI people, discrimination and hostility have been reflected in the “moral panic” (152) and “cultural bullying” (130), associated with political and media condemnation of initiatives addressing LGBTQI bullying in primary and secondary schools (152), or the right for trans and non-binary people to participate in sport (153). There has been widespread media coverage of “homosexual acts” being associated with bestiality, incest and pedophilia (154), or with abusive relationships (155) and the insistence transgender students identify as “the gender that God bestowed” (154). Prejudicial LGBTQI public discourse is often accompanied by discriminatory practices in healthcare (109) and the workplace (156, 157) as well as acts of hate speech and violence (112, 158) in both Australia, the USA and other international contexts where our participants resided.

This cultural milieu of hostility towards LGBTQI people is reflected in the finding that the majority of participants in the present study (84%) reported experiences of anti-LGBTQI discrimination at some point in their lives. These rates are higher than previously reported for sexual minority breast cancer survivors in the USA, using similar measurement tools (48%) (43). A further 33% had experienced discrimination as part of their cancer care, which is higher than most rates (2-41%) reported in previous research on discrimination in LGBTQI general healthcare (109). Oncology health care professionals report a lack of knowledge and confidence in treating LGBTQI patients (159, 160), in particular patients who are TGD or have an intersex variation (79), which can lead to levels or forms of care that are not LGBTQI inclusive, including inappropriate comments, exclusion of partners and hostility (26). Previous research has demonstrated that inappropriate comments, hostility and discriminatory practice on the part of health care professionals was associated with negative psychological and physical outcomes for LGBTQI people (109, 161), including LGBTQI people with cancer (2, 64). These findings are confirmed in the present study, with the mechanisms of this effect including cis-heteronormative health care professional practices, hostility toward LGBTQI patients and their carers, and a lack of LGBTQI cancer information. This has been explored further in the qualitative arm of the *Out with Cancer Study* (26, 60).

Social support has also been demonstrated to be associated with better QOL and functioning in the general cancer population (70). Social support can also reduce the negative impact of minority stress (43), through buffering or protecting against stress (162), explored in the qualitative arm of the *Out with Cancer Study* (60). In the present study, social support was negatively correlated distress and positively correlated with QOL for LGBTQI people with cancer. This confirms previous reports that low social support was associated with distress in lesbians with breast cancer (43, 163, 164) and gay/bisexual men with prostate cancer (165, 166), validating the argument that absence of social safety is a fundamental cause of mental and physical health disparities in LGBTQI populations (78). It has been reported that many LGBTQI individuals report sustained social isolation because of cancer (166, 167). In the non-LGBTQI community the primary carers of adults with cancer are typically their intimate partners (70), whereas LGBTQI individuals often look for support through broader social support networks and communities. For example, in a recent study of Australian gay men with prostate cancer, 39% were partnered (9), compared with 61% of the general population of the same age (55). However, social support is high in the present study, comparable to or higher than social support reported in the non-LGBTQI people with cancer (168–170), with the majority of participants reporting a range of supportive networks, including intimate partners, friends, other LGBTQI people, family and colleagues. These findings confirm previous reports that ‘chosen family’ and LGBTQI communities provide social support and connectedness for older LGB people (62, 73, 74, 171). TGD, intersex, AYA, queer and bisexual sub-groups in this study report significantly lower levels of social support, in line with previous findings that people who identify as queer, transgender, or genderqueer reported lower support than other SGM people with cancer (172). There is no previous research examining social support in AYA or intersex LGBTQI people with cancer. It is widely recognized that family support and acceptance is a protective factor for the mental health and wellbeing of LGBTQ AYAs in the general population (75, 128), alongside quality relationships with friends (173). Further research is needed to systematically examine the interactive effects of social support and psycho-social variables associated with distress and QOL for LGBTQI cancer survivors, to determine if social support reduces negative effects.

### 4.1 Study limitations

There are several limitations to the present study. It is a cross sectional study, with a small sample size in some subgroups. Further research is needed including larger numbers of AYA, TGD, bisexual and queer subgroups. Longitudinal research to examine experiences of LGBTQI cancer survivorship would also be useful. A further limitation is the use of truncated measures for some indices, due to the wide range of indices examined in this exploratory study, and the use of unvalidated measures where validated measures developed for the general cancer population were not appropriate for LGBTQI communities. Future research should use expanded and validated scales and validate existing scales for the LGBTQI population. The study may have been affected by sampling and self-report biases. As participants responded to invitations to take part in the online survey, the sample may not be representative of all LGBTQI people with cancer, particularly those who have limited digital literacy or access to technology, or who were not members of the platforms or organizations through which the survey was advertised. A further limitation is that the study relies on self-reported cancer diagnosis collected by anonymous survey methods. However, as LGBTQI status is not recorded by most cancer registries and hospital clinics, participants could not be accessed through medical records.

### 4.2 Conclusion

Our findings add further insight into the mechanisms of negative psycho-social outcomes for LGBTQI cancer patients and survivors, highlighting the impact of minority stress and the buffering effects of social support, and identifying diversity within LGBTQI populations related to health outcomes (1, 28). Those who are TGD, who have a variation in sex characteristics, who identify as queer or bisexual, and younger LGBTQI people with cancer, may be more vulnerable to distress and low QOL. However, these sub-groups of individuals are not independent identity positions that can be considered separately from each other (82). A person may be multiply marginalized due to their gender, their sexuality, their intersex status and their age, in what has been described as a double or triple jeopardy, within a “both/and” framework (29, 82). Equally, the social meaning and power relationships inherent in sexuality, gender identity, age and intersex status cannot be considered separately from each other (82). Our multiple comparison points thus reflect intersecting identities and vulnerabilities, suggesting a “matrix of domination” (174) in which multiple marginalized identities (29), based on social or LGBTQI sub-group membership, intersect to create life situations and psychological outcomes that are qualitatively different depending on one’s location in the matrix (82). There is a need for further research to examine the ways in which intersecting identities and stressors operate to produce both positive and negative psycho-social outcomes for LGBTQI people with cancer, using both qualitative and quantitative methods. Further research is also needed to examine the intersection of cultural background and ethnicity with LGBTQI status. This was not possible in the present study, due to the small number of participants who did not identify as white/Caucasian and the disparities in background in the non-white/Caucasian grouping.

Our findings reinforce the conclusion of The American Society of Clinical Oncology (6) that it is imperative that attention is paid to health disparities experienced by LGBTQI people with cancer. Oncology research needs to include measures of sexuality and gender diversity, and intersex variation, as a matter of course, to avoid rendering invisible this potentially vulnerable group of patients and survivors and to identify unmet needs in LGBTQI experiences of cancer and cancer care. More information is needed about the unique experience of LGBTQI cancer patients, survivors and their carers, with a particular focus on the overlooked and intersecting groups of TGD, intersex and AYA people. Co-design of research and collaboration with LGBTQI stakeholders can help to ensure the LGBTQI cultural competence and cultural safety of methods and interpretation (175).

It is essential that we develop inclusive and affirmative cancer care for LGBTQI patients (176), including content related to the needs and experiences of the LGBTQI community overall, as well as content specific to each sub-group (79). Practical initiatives start with provision of LGBTQI content in health care professional education and training curricula to facilitate understanding of this often-overlooked population in cancer care and to challenge bias and ingrained cis-heteronormative practices (26, 159, 176). Specific practices to develop inclusive and affirmative LGBTQI cancer care include: avoiding the assumption that patients are heterosexual and cisgender by asking what patients prefer as names and pronouns; not making assumptions about the patients’ relationships with the persons accompanying them to appointments; including same-gender partners in care; not assuming only heterosexual cisgender people want to discuss sexual health and fertility concerns; and encouraging LGBTQI patients to connect with peers (2, 176–178).

In order to be LGBTQI inclusive, cancer centers, hospitals and cancer community organizations should display LGBTQI images and logos, provide gender neutral bathrooms, tailored LGBTQI-inclusive supportive resources, and include LGBTQI people in general cancer information (2, 79, 159, 176). Services need to be accountable through formal mechanisms for addressing complaints about discrimination and poor care, which includes clear information about complaints processes for patients, and taking such complaints seriously. Intake forms should include sexuality, gender identity, preferred name and pronoun and intersex variation (159, 176), in order to facilitate LGBTQI patient disclosure (179). In combination, these measures will increase the likelihood of the needs of LGBTQI people with cancer being acknowledged and met, resulting in non-discriminatory and inclusive cancer care for LGBTQI patients and their carers, with positive implications for patient health outcomes.

## Data availability statement

The raw data supporting the conclusions of this article will be made available by the authors, without undue reservation.

## Ethics statement

The studies involving human participants were reviewed and approved by Western Sydney University Human Research Ethics Committee. All participants provided written informed consent to participate in this study.

## Author contributions

JU and JP designed the study and prepared the application for funding, in collaboration with The Out with Cancer Study team members. The survey was developed by JU, KA, and RP in collaboration with the Out with Cancer Study team, and our stakeholder advisory group. Data were collected by RP and KA. KA conducted statistical analysis of the data, in collaboration with JP and JU. JU and KA wrote the paper, with critical input from JP and RP. The Out with Cancer Study Team provided critical commentary on the written paper. All authors contributed to the article and approved the submitted version.

## The Out with Cancer Study team members involved in this paper

Chloe Parton^1^, Alexandra Hawkey^2^, Gary W. Dowsett**^3^** Fiona E. J. McDonald^4^, Antoinette Anazodo^5^, Suzanne Chambers^6^, Martha Hickey^7^, Kerry H. Robinson^8^, Felix Delhomme^9^ Scout^10^ and Katherine Boydell^11^

^1^ School of Health, Te Herenga Waka – Victoria University of Wellington, Wellington, New Zealand

^2^ Translational Health Research Institute, Western Sydney University, Sydney, Australia

^3^ Australian Research Centre in Sex, Health and Society, La Trobe University, Melbourne, Australia

^4^ Canteen and Faculty of Medicine and Health, The University of Sydney, Sydney, Australia

^5^Kids Cancer Centre, Sydney Children’s Hospital and School of Women’s and Children’s Health, University of New South Wales, Sydney, Australia

^6^ Faculty of Health Sciences, Australian Catholic University, Brisbane, Australia

^7^Department of Obstetrics and Gynaecology, University of Melbourne and the Royal Women’s Hospital, Melbourne, Australia

^8^ School of Social Sciences and Translational Health Research Institute, Western Sydney University, Sydney Australia

^9^ACON, Sydney, Australia

^10^National LGBT Cancer Network, USA

^11^ Black Dog Institute, University of New South Wales, Sydney, Australia

## Funding

The *Out with Cancer Study* was funded by the Australian Research Council Linkage Program grant [LP170100644], the Cancer Council New South Wales, and Prostate Cancer Foundation Australia, with in-kind support provided by National LGBTI Health Alliance, ACON, Breast Cancer Network Australia, Sydney Children’s Hospital Network, and Canteen. The chief investigators of the project were Jane Ussher, Janette Perz, Martha Hickey, Suzanne Chambers, Gary Dowsett, Ian Davis, Kerry Robinson, Chloe Parton. Partner Investigators: Antoinette Anazodo, Fiona MacDonald

## Acknowledgments

We acknowledge Samantha Ryan, Jack Thepsourintheone, Samantha Sperring and Colin Ellis for assistance in data collection. We thank our stakeholder advisory board for their input into the project. This research was supported by ANZUP and by Register4 through its members’ participation in research. We would also like to thank all our LGBTQI participants who volunteered for this study.

## Conflict of interest

The authors declare that the research was conducted in the absence of any commercial or financial relationships that could be construed as a potential conflict of interest.

## Publisher’s note

All claims expressed in this article are solely those of the authors and do not necessarily represent those of their affiliated organizations, or those of the publisher, the editors and the reviewers. Any product that may be evaluated in this article, or claim that may be made by its manufacturer, is not guaranteed or endorsed by the publisher.

## Footnotes

1 Chi-square tests indicated that there were no significant differences in the likelihood of being partnered by gender, sexuality, intersex status, age, geographic location or cancer type, after Bonferroni correction. 

## Appendix

**Table A1:** Effect sizes and tests of difference for distress and quality of life (by gender, sexuality, intersex status, age, cancer type).

	Gender			Sexuality			Intersex status			Age			Remoteness*			Cancer type		
	η^2^	*F*	*p*	η^2^	*F*	*p*	η^2^	*F*	*p*	η^2^	*F*	*p*	η^2^	*F*	*p*	η^2^	*F*	*p*
Distress	.044	7.084	**<.001**	.051	8.095	**<.001**	.021	6.662	.010	.092	31.959	**<.001**	.034	5.557	**.004**	.010	2.782	.096
QOL	.069	12.167	**<.001**	.074	12.718	**<.001**	.048	16.360	**<.001**	016	5.311	.022	.007	1.206	.301	.005	1.319	.252

Note: statistically significant p-values (p≤.008, using Bonferroni correction) are indicated in bold text. *Urban = in a major city of 100,000+ people, or the surrounding suburbs; Regional = in a smaller city; Rural = outside of a city. QOL = quality of life.

**Table A2:** Effect sizes and tests of difference for other study variables (by gender, sexuality, intersex status, age and cancer type).

	Gender			Sexuality			Intersex status			Age			Remoteness*			Cancer type		
	η^2^	*F*	*p*	η^2^	*F*	*p*	η^2^	*F*	*p*	η^2^	*F*	*p*	η^2^	*F*	*p*	η^2^	*F*	*p*
Sexual concerns	.014	1.852	.159	.003	0.351	.704	.027	7.433	**.007**	.000	0.076	.783	.000	0.038	.962	.022	5.261	.023
Physical concerns	.018	2.645	.073	.052	7.993	**<.001**	.000	0.003	.958	.033	10.235	**.002**	.010	1.479	.229	.006	1.445	.230
LGBTQI impact	.044	9.308	**<.001**	.010	1.952	.143	.002	0.764	.383	.008	3.232	.073	.001	0.188	.829	.005	1.835	.176
Gender impact	.144	27.245	**<.001**	.120	21.586	**<.001**	.018	5.757	.017	.028	9.535	**.002**	.001	0.222	.801	.001	0.384	.536
FCR	.002	0.355	.702	.011	1.768	.172	.004	1.278	.259	.019	6.322	.012	.001	0.218	.804	.003	0.971	.325
Minority stress																		
Discr (*gen*)	.062	13.476	**<.001**	.014	2.840	.060	.032	13.556	**<.001**	.019	7.876	**.005**	.003	0.545	.580	.003	1.251	.264
Discr (*care*)	.073	15.886	**<.001**	.032	6.556	**.002**	.064	27.439	**<.001**	.018	7.413	**.007**	.002	0.377	.714	.000	0.035	.851
Discomf LGBTQI	.043	9.066	**<.001**	.082	17.493	**<.001**	.018	7.352	**.007**	.057	24.698	**<.001**	.001	0.233	.792	.004	1.412	.236
Outness	.057	7.448	**<.001**	.221	54.461	**<.001**	.008	2.350	.126	.091	39.800	**<.001**	.006	1.275	.281	.002	0.448	.504
Social support	.046	7.448	**<.001**	.036	6.577	**.002**	.025	9.338	**.002**	.025	9.585	**.002**	.005	0.865	.422	.000	.002	.966

Note: statistically significant p-values (p≤.008, using Bonferroni correction) are indicated in bold text. *Urban = in a major city of 100,000+ people, or the surrounding suburbs; Regional = in a smaller city; Rural = outside of a city. Discomf LGBTQI = discomfort being LGBTQI; discr (care) = discrimination in cancer care; discr (gen) = discrimination in general life; FCR = fear of cancer recurrence; QOL = quality of life.

**Table A3:** Chi-square analyses exploring differences within groups (by gender, sexuality, intersex status, age, remoteness and cancer type) in correlations between quality of life and other study variables.

Variable	Gender	Sexuality	Intersex status	Age	Remoteness**	Cancer type
Sexual concerns	2.456	3.211	0.128	0.027	0.126	0.275
Physical concerns	0.032	2.923	0.502	0.324	0.612	2.153
LGBTQI impact	0.570	3.363	0.191	1.107	0.218	0.361
Gender impact	**10.806***	1.450	0.679	0.116	1.202	0.724
FCR	0.196	2.573	1.222	4.489	4.627	1.489
Minority stress						
Discr (*gen*)	1.730	5.546	0.013	3.344	2.884	4.910
Discr (*care*)	0.315	2.344	2.440	6.163	2.290	0.002
Discomf LGBTQI	0.361	0.064	2.304	0.133	0.099	1.306
Outness	4.112	1.419	0.061	0.250	0.980	1.252
Social support	2.776	4.871	0.556	1.148	1.059	0.192

*Bold text denotes chi-square values which are statistically significant (p≤.008, using Bonferroni correction). **Urban = in a major city of 100,000+ people, or the surrounding suburbs; Regional = in a smaller city; Rural = outside of a city. AYA = adolescent and young adult (15-39 years); B = bisexual; CF = cis female; CM = cis male; Discomf LGBTQI = discomfort being LGBTQI; Discr (care) = discrimination in cancer care; Discr (gen) = discrimination in general life; FCR = fear of cancer recurrence; LG = lesbian/gay; Non = non-reproductive; Q = queer; Reg = regional; Repr = reproductive

**Table A4:** Chi-square tests exploring differences within groups (by gender, sexuality, intersex status, age, remoteness and cancer type) in correlations between distress and other study variables.

Variable	Gender	Sexuality	Intersex status	Age	Remoteness**	Cancer type
Sexual concerns	1.381	8.506	0.423	0.014	1.701	0.532
Physical concerns	0.084	0.640	3.764	0.000	1.474	0.770
LGBTQI+ impact	1.448	0.940	0.950	2.733	0.803	0.612
Gender impact	3.622	0.192	0.042	0.268	5.147	3.051
FCR	1.151	0.190	0.601	0.029	9.392	0.006
Minority stress						
Discr (*gen*)	2.851	4.506	0.024	0.018	4.252	2.914
Discr (*care*)	0.695	1.474	3.724	0.953	0.457	0.287
Discomf LGBTQI	1.057	0.484	0.001	0.369	1.032	0.484
Outness	0.346	6.049	0.023	0.099	0.510	0.330
Social support	4.382	1.623	**12.546***	0.001	2.059	1.643

*Bold text denotes z-scores which are statistically significant (p≤.008, using Bonferroni correction). **Urban, in a major city of 100,000+ people, or the surrounding suburbs; Regional, in a smaller city; Rural, outside of a city. AYA, adolescent and young adult (15-39 years); B, bisexual; CF, cis female; CM, cis male; Discomf LGBTQI, discomfort being LGBTQI; Discr (care), discrimination in cancer care; Discr (gen), discrimination in general life; FCR, fear of cancer recurrence; LG, lesbian/gay; Non, non-reproductive; Q, queer; Reg, regional; Repr, reproductive.
